# Multiple Endocrine Neoplasia with Pulmonary Localization: A New Protocol of Approach

**DOI:** 10.1100/tsw.2008.103

**Published:** 2008-08-08

**Authors:** Duilio Divisi, Sergio Di Tommaso, Giovanna Imbriglio, Roberto Crisci

**Affiliations:** Department of Thoracic Surgery, University of l'Aquila, “G. Mazzini” Hospital, Teramo, Italy

**Keywords:** neuroendocrine tumors, multiple endocrine neoplasia, surgical treatment

## Abstract

We present three patients with bronchial carcinoids, in which a more probed study emphasized the presence of three multiple endocrine neoplasia (MEN). Assessment included a total-body computerized tomography, a total-body single-photon emission computerized tomography by In-DTPA-D-Phe octreotide, and genetic map. Two patients presented an atypical MEN 1 and one patient showed an atypical MEN 1 with a familial medullary thyroid carcinoma. All patients were operated upon: two are still alive and one died 50 months after the first intervention. Precocious diagnosis of MEN permits a good long-term outcome.

## INTRODUCTION

Multiple endocrine neoplasia (MEN), transmitted by an autosomal-dominant pattern of inheritance, represent a rare variation of neuroendocrine tumors. MEN show the presence of various endocrine neoplasms in the same patient that can appear either at the same time, at a distance of time, or never be visible. Clinical aspects are variable and complicated due to the production of the biologically active substances. There are two major forms: MEN 1 and MEN 2[1,2]. MEN type 1 is characterized by parathyroid, pancreatic islet, and anterior pituitary tumors; gastrointestinal and lung neoplasms or suprarenal adenomas can be found. MEN type 2 presents these subgroups: (1) MEN 2A, characterized by medullary thyroid carcinoma, pheochromocytoma, and primary hyperparathyroidism; (2) MEN 2B, characterized by medullary thyroid carcinoma, pheochromocytoma, marfanoid habitus, and ganglioneuromatosis; (3) FMTC, in which medullary carcinoma appears alone.

Our experience refers to this complicated topic through the study of three patients with the common characteristic of the presence of bronchial carcinoids.

## CASE 1

A 28-year-old woman came to our observation with hemoptysis. Chest X-ray revealed a lesion in the right pulmonary parenchyma. Total-body computerized tomography (CT) highlighted a 4-cm diameter mass located in the middle pulmonary lobe and a 2-cm pancreatic neoformation with clear outlines. Fiber-optic bronchoscopy allowed diagnosis of typical carcinoid. Total-body single-photon emission computerized tomography (SPECT) by $^{111}$In-DTPA-D-Phe$^{1}$ octreotide confirmed the localizations cleared at CT. Serum levels of neuron-specific enolase (NSE) and chromogranin A (CgA) were positive; urinary level of 5-hydroxy-3-indoleacetic acid (5-HIAA) was negative. Immunohistochemistry was positive for CgA and synaptophysin. The patient underwent middle lobectomy and pancreasectomy; postoperative histological evaluation was of typical bronchial carcinoid ($T_1N_0M_0$: Stage IA) and pancreatic insular tumor type gastrinoma with G nonsecreting cells. In consideration of these characteristics, the anamnesis and a clinical-functional study of parathyroid and suprarenal glands were carried out; results were also normal. Genetic map revealed a deletion of three a.a. on exons 6 and 8 of oncosuppressor gene MEN 1. After 7 years from the interventions, the patient shows excellent health, without recurrence of pathology. On the basis of the clinical and surgical history, a final diagnosis of atypical multiple neuroendocrine syndrome type MEN 1 was expressed.

## CASE 2

A 54-year-old woman came to the emergency room with acute sight disorders and a persistent headache. Total-body CT showed a 3.5-cm diameter lesion located in the hypophysis. Intraoperative histological diagnosis was of inactive pituitary macroadenoma, with consequent excision and complete resolution of the symptomatology. One year and 3 months later, the patient came to our observation with hemoptysis. Total-body CT highlighted an atelectasis of the right superior pulmonary lobe. Fiber-optic bronchoscopy revealed a smooth red neoformation in the right upper lobe bronchus; multiple biopsies allowed diagnosis of atypical carcinoid. Total-body SPECT by $^{111}$In-DTPA-D-Phe$^{1}$ octreotide confirmed a singular localization in the thorax. Serum level of NSE (0–15.2 μg/ml) and CgA (60 ηg/ml) was positive as was the immunohistochemical assessment of CgA and synaptophysin. Patient underwent superior right lobectomy with a histological evaluation of atypical bronchial carcinoid without lymph node metastases ($T_2N_0M_0$: Stage IB). Anamnesis revealed that the 60-year-old brother underwent lung excision for carcinoma. Functional study of parathyroid and suprarenal glands gave normal results. Genetic map highlighted an alteration on exon 8 of oncosuppressor gene MEN 1. Four years from intervention, the patient shows excellent health, without neuroendocrine syndrome. Clinical and surgical history suggested a definitive diagnosis of atypical multiple neuroendocrine syndrome type MEN 1.

## CASE 3

A 44-year-old man came to our observation because of a hemoptysis and persistent cough. Total-body CT highlighted a 4.5-cm diameter neoformation located in the inferior right pulmonary lobe with clear outlines (Fig. 1). Fiber-optic bronchoscopy revealed a smooth vegetation in the right lower lobe bronchus; biopsies allowed diagnosis of typical carcinoid. Total-body SPECT by $^{111}$In-DTPA-D-Phe$^{1}$ octreotide confirmed the thorax localization. Serum level and immunohistochemistry of tumor markers were positive. Patient underwent inferior right lobectomy for typical bronchial carcinoid without lymph node metastases ($T_2N_0M_0$: Stage IB). One year later, the patient was hospitalized owing to visual field illness. Total-body CT and SPECT by $^{111}$In-DTPA-D-Phe$^{1}$ octreotide showed a 2.5-cm diameter lesion located in the hypophysis (Fig. 2). Intraoperative histological diagnosis was of inactive pituitary macroadenoma; excision allowed resolution of the symptomatology. Two years after the second intervention, the patient carried out a routine control examination that checked the presence of a palpable nodule in the left thyroid lobe. CT of the neck and a total-body SPECT by $^{111}$In-DTPA-D-Phe$^{1}$ octreotide showed a circular left thyroid lesion with diameter of 2.5 cm (Fig. 3). Calcitonin levels were elevated to 650 pg/ml. Fine needle aspiration biopsy (FNAB) permitted diagnosis of medullary thyroid carcinoma. Patient underwent total thyroidectomy for stage II medullary thyroid carcinoma. Anamnesis showed that the father and the great

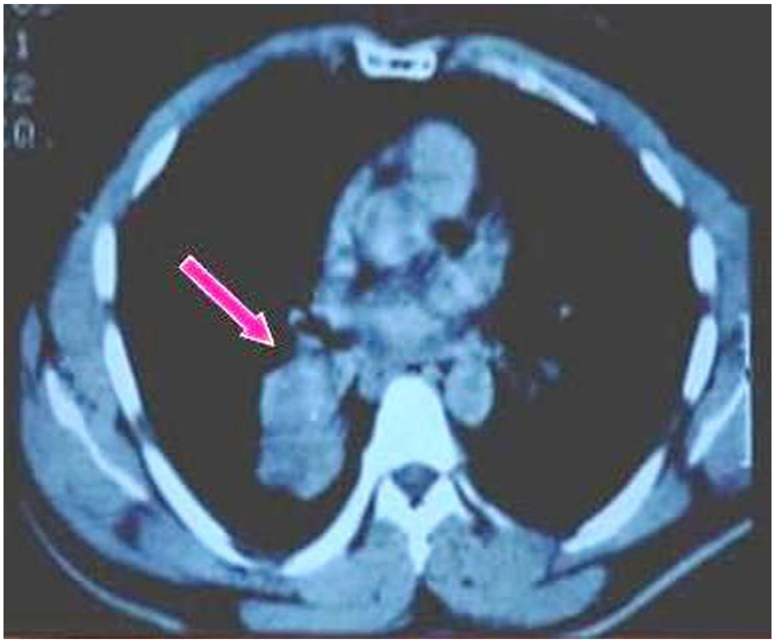

**FIGURE 1.** Oval lesion in the right lower lobe.

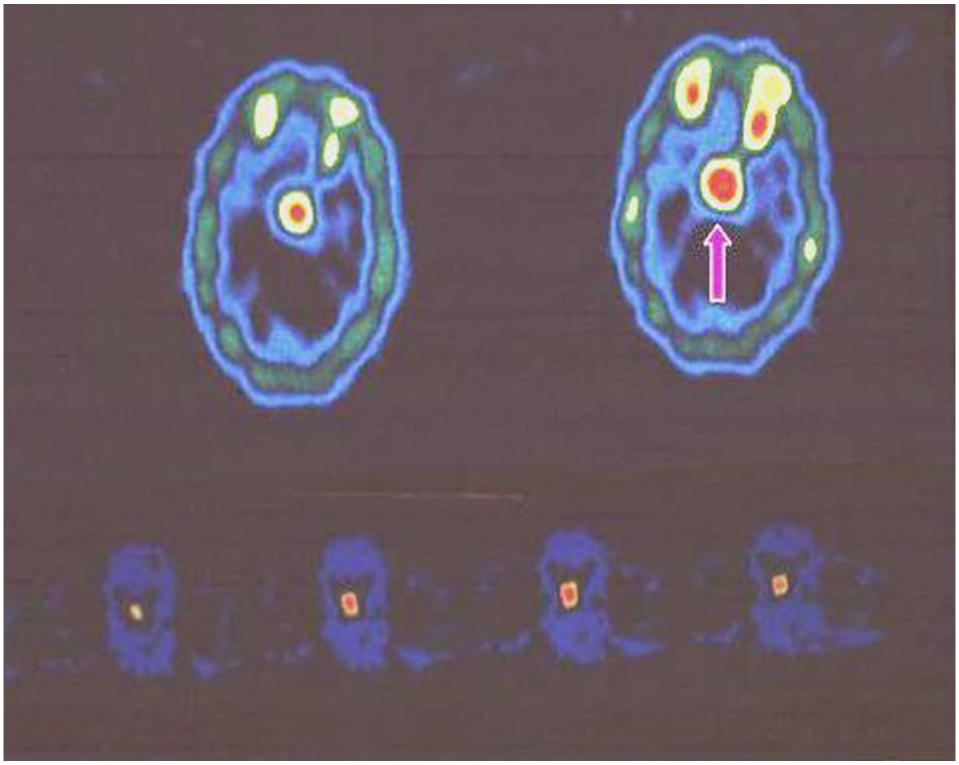

**FIGURE 2.** Octreotide scintigraphy of pituitary neoformation.

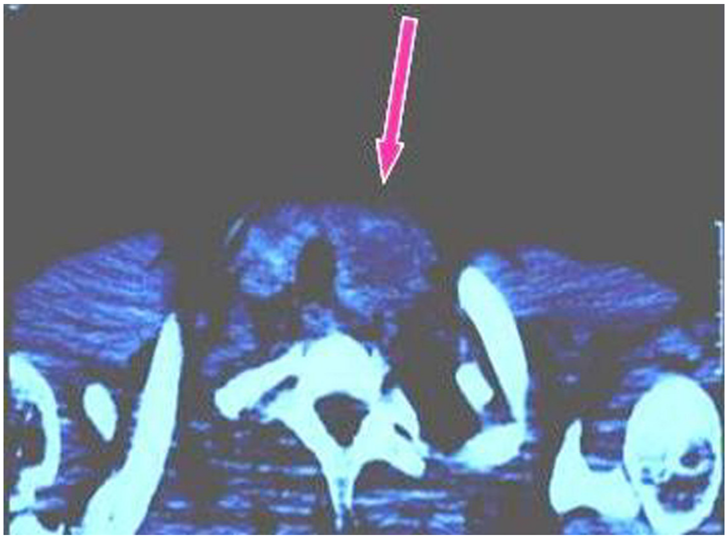

**FIGURE 3.** Tomography of left thyroid mass.

uncle had thyroid disease. Functional study of parathyroid and suprarenal glands was negative. Genetic map highlighted a site-splice alteration on exon 6 of oncosuppressor gene MEN 1 and proto-oncogene RET missense mutation. On the basis of the clinical and surgical history, a final diagnosis of atypical multiple neuroendocrine syndrome type MEN 1 associated to FMTC was expressed. Eight months later, the patient showed a bone metastases (right femur, ribs and cranial). He was treated with somatostatin analog (octreotide), but passed away after 6 months because of cardiac failure.

## DISCUSSION

Our experience underlined the necessity of an accurate anamnesis and a total-body CT and SPECT by $^{111}$In-DTPA-D-Phe$^{1}$ octreotide in the multiple endocrine syndrome. MEN 1 is linked to germline mutations on the MEN 1 tumor suppressor gene located on the long arm of chromosome 11 (11q13)[3]. Germline mutations on the proto-oncogene RET located on the long arm of chromosome 10 (10q11.2) are responsible for MEN 2[4]. A genetic map of the patient and his/her family can show unrecognized inherited diseases and presymptomatic diagnosis. In fact, the simple alteration of genetic screening in MEN 2 authorizes the early prophylactic thyroidectomy. CT allows the primary identification of lesions and the evaluation of topographic and morphological aspects; neoformations smaller than 1 cm cannot be distinguished with CT. SPECT by $^{111}$In-DTPA-D-Phe$^{1}$ octreotide defines the exact localization and stage of tumor owing to the high affinity to sst2 and the low affinity to sst3 and sst5 subtype receptors of somatostatin; this device orientates the surgical approach. Physiological characteristics of octreotide (long half life of the tissue and rapid plasmatic clearance) explain the higher specificity and sensitivity with SPECT by $^{111}$In-DTPA-D-Phe$^{1}$ for the diagnosis of MEN, lymph node invasion, and repeat phenomena. Shi et al.[5], in a comparative study of SPECT vs. CT and magnetic resonance imaging (MRI), show unexpected mass in 40 sites not detected by conventional techniques and defined by octreoscan in 50% of MEN patients. Complexity of neuroendocrine tumors make the use of chemotherapy still debatable[6]. Octreotide is a valid alternative because it inhibits the growth factors on the tumor cells and increases the binding protein IGF-I. This method must be applied in the case of metastases, clinical intolerance of complementary treatment, or chemoresistance. The role of surgery is uncontroversial, determining the pathological stage of the tumors and long-term survival. Our study found the rare presence of pulmonary localization and the unusual association between MEN 1 and FMTC. A bone relapse after many years in one patient highlights the low oncological aggressiveness of the neoplasms. The surgical approach must ensure histological completeness. The indications of excisions have already been discussed and established in bronchial carcinoids[7]. Nikou et al.[8], in Zollinger-Ellison and MEN 1 syndrome patients, treated only 54% of pancreatic or duodenal gastrinomas surgically and the associated parathyroid adenomas in all patients, pituitary adenomas in three patients, and bronchial carcinoid in one patient. Survival rate was 91%.

## CONCLUSIONS

The pathogenesis and clinical features of MEN need a protocol of study based on genetic, radiological, functional analysis, and follow-up of the different organs potentially involved in the MEN. This behavior permits an effective precocious diagnosis and surgical treatment, improving the prognosis of patients. In the future, the progress of genetic-molecular techniques will make it easier to classify MEN patients, with a nearly complete possibility of healing.
